# Evaluating the Efficacy and Complications of the Scapular Osseous Free Flap for Head and Neck Reconstruction: Results from a Population-based Cohort

**DOI:** 10.1016/j.jpra.2024.09.020

**Published:** 2024-10-01

**Authors:** Henrik Guné, Johanna Sjövall, Magnus Becker, Karin Elebro, Anna Hafström, Linda Tallroth, Stina Klasson

**Affiliations:** a Department of Plastic and Reconstructive Surgery, Skåne University Hospital, Malmö, Sweden; b Department of Otorhinolaryngology-Head and Neck Surgery, Skåne University Hospital, Lund, Sweden; c Department of Clinical Sciences, Lund University, Lund, Sweden

**Keywords:** Scapular free flap, Head and neck cancer, Microsurgery, Reconstructive surgery, Maxillary reconstruction, Mandibular reconstruction

## Abstract

**Introduction:**

The scapular osseous free flap (SOFF) is being increasingly used for complex head and neck reconstructions. This study examined the surgical outcomes, focusing on post-operative complications and sequelae in patients who underwent SOFF for maxillary and mandibular reconstructions.

**Material and Methods:**

This retrospective, observational, population-based study included patients who underwent SOFF reconstruction at a tertiary referral centre, the Department of Otorhinolaryngology-Head and Neck Surgery, Skåne University Hospital, Sweden, from November 2016 to March 2023. All patients were followed-up for at least six months after surgery.

**Results:**

Forty-two of the 44 consecutive patients (60 % men) consented to the study and were evaluated with a median follow-up time of 49 months (range 8–85 months). The study divided the patients into two groups; maxillary (*n* = 29) and mandibular (*n* = 13) reconstructions. The World Health Organisation performance status and the Charlson comorbidity index were lower in the maxillary group (*p* = 0.025 and *p* = 0.011, respectively). The maxillary group experienced high complication rates including six total flap failures and nine oronasal fistulas. Conversely, the mandibular group had no flap failures but a similar rate of general post-operative complications were observed. Dental rehabilitation was more common in the maxillary group.

**Conclusion:**

The SOFF is an option for complex reconstructions of the maxilla but is associated with a relatively high rate of complications. Methods that can prevent or minimise sequelae, e.g., oronasal fistulas, in future patients are warranted. The SOFF is an excellent alternative for mandibular reconstructions.

## Introduction

The scapular osseous free flap (SOFF) was introduced in the 1980s as an option for maxillary and mandibular reconstructions and has relatively recently increased in popularity.^1^, ^2^, ^3^, ^4^ The need for adequate pedicle length has led to the use of the angular branch of the thoracodorsal artery.^2^^,^^5^^,^^6^

The chimeric nature of the subscapular vascular system, allows the harvest of skin paddles, serratus muscle with or without a rib, latissimus dorsi (LD) muscle and up to 14 cm of scapular bone gives SOFF several advantages over the fibula free flap (FFF) and iliac crest free flap in the reconstruction of palatomaxillary defects.^5^^,^^7^

The FFF has been the first-choice flap for long mandibular defects. However, the SOFF is an option for reconstructing mandibular defects as the subscapular vessels are less affected by atherosclerosis and the donor site morbidity is limited.^5^^,^^8^^,^^9^

Only a few studies have been published on the outcomes of SOFF in maxillary reconstruction but several post-operative problems have been described. One of the more troublesome is the formation of an oronasal fistula which is reported in 4–21 % of the maxillary reconstructions.^7^^,^^10^^,^^11^

In the Southern Health Care Region of Sweden, with a catchment area of approximately 1.9 million inhabitants, all patients with head and neck malignancies are treated and followed-up at the Skåne University Hospital, a tertiary referral hospital.

The present study aims to present the surgical outcomes in a population-based cohort of consecutive patients with regard to post-operative complications and sequelae following maxillary and mandibular reconstruction with SOFF.

## Materials and methods

### Study design and cohort

This was a retrospective, observational, population-based study carried out with ethical approval. All patients who underwent SOFF reconstruction at the Dept. of Otorhinolaryngology-Head and Neck Surgery at Skåne University Hospital, Sweden from November 2016 to March 2023 were invited to participate in the analysis. All patients were followed-up for at least six months after the surgery. The patients were divided into two groups, patients undergoing maxillary reconstruction and those undergoing mandibular reconstruction.

### Criteria for choosing SOFF in maxillary and mandibular reconstruction

SOFF was chosen in patients with extensive maxillary defects, Brown classification II-IV,^12^ where dental implants were warranted. Patients, with significant comorbidities or only posterior palatal defects, were recommended soft tissue free flap.

For mandibular defects, SOFF reconstruction was the primary option in patients with arteriosclerosis, abnormal anatomy or trauma of the lower limb and chosen after individual assessment in elderly patients.

### Demographic and medical history

Demographic and clinical data were collected from medical charts along with study specific information such as comorbidities, smoking history, tumour histopathology and T-classification stage as well as type of midface resection and previous (c) RT for head and neck malignancy. Survival data were obtained from the Swedish Population Register.

### Definitions

Malignant tumours were staged according to the International Union Against Cancer (UICC) tumour-node-metastasis (TNM) classification, 8th edition.^13^

A current smoker had smoked any number of cigarettes within the previous six months, and a previous smoker had stopped smoking at least six months previously. Maxillectomy defects were classified at time of surgery using the classification system developed by Brown et al.^12^ The osseous SOFF flap is defined as scapular bone and part of the teres major muscle. Performance status was assessed according to WHO performance status. The Charlson comorbidity index (CCI) was assessed.^14^ General complications were categorised using the Clavien–Dindo classification for surgical complications.^15^ A surgical takeback was defined as a return to the operating theatre during the post-operative hospitalisation period. Facial or donor site infections were identified as prolonged or re-administration of antibiotic treatment. Aesthetic outcomes included over- or under contoured facial features, ectropion, globe malposition and inadequacy of the gingivobuccal sulcus were evaluated as either present or absent. The assessment was conducted six to 12 months after surgery and involved a collaborative judgement between a senior head and neck surgeon and a senior plastic surgeon during the post-operative follow-up.

### Surgical techniques and procedures

Tumour resection and reconstruction were performed within a single surgical procedure. When needed, the Weber Fergusson (WF) approach with a sub ciliary incision was used. The technique of flap harvest and reconstruction of maxillectomy defects were carried out as previously described by Clark et al.^2^

The primary tumour resection was performed simultaneously as the SOFF flap was harvested from the ipsilateral side to the tumour resection.

According to local procedure traditions, the SOFF vessels were anastomosed before the inset of the flap. A Cook-Swartz Doppler probe® (Cook Group, Inc., Indiana, USA) was connected to the thoracodorsal artery for monitoring. The inset of the bony part of the flap was performed by a maxillofacial surgeon. Soft tissue reconstruction was then carried out in collaboration with the plastic surgeon and head and neck surgeon.

### Standard perioperative care

All patients spent the first post-operative night in the intensive care unit (ICU) and received the first dose of low molecular weight heparin the night before surgery (enoxaparin, 4000 IE (40 mg)/0.4 ml) then twice daily during the hospitalisation period. Prophylactic antibiotics, cefotaxime and metronidazole, were administered intravenously for three days. A nasogastric feeding tube was applied preoperatively, and oral liquid intake was permitted 4–5 days post-operatively. Rehabilitation started on the second day after surgery with scapula-specific physiotherapy.

### Statistical analysis

Categorical data were given as proportions and continuous variables expressed as median and range (min-max). The groups were compared using the chi-squared test and if the expected frequency was <5, the Fisher's exact test was applied. For continuous variables, the Mann–Whitney U test was used. A *p*-value <0.05 was considered statistically significant. Statistical analysis was performed on standard software (IBM Corp. Released 2017. IBM SPSS Statistics for Mac, Version 25. Armonk, NY: IBM Corp).

## Results

### Study population and patient follow-up

Out of 44 eligible patients, two patients declined participation, leaving 42 individuals, 25 men and 17 women, eligible for analysis, which was performed in November 2023. The median follow-up time was 49 months (range 8–85 months). Twenty-nine patients had maxillary and 13 patients had mandibular reconstructions.

### Preoperative and surgical characteristics

The median age for all patients was 63 years (range 22–78 years). All patients, except for two in each group (maxillary and mandibular), had the SOFF because of malignant tumours (Table 1). The histopathologies included squamous cell carcinoma (*n* = 25), adenocarcinoma (*n* = 6), adenoid cystic cancer (*n* = 3), mucosal malignant melanoma (*n* = 2), osteosarcoma (*n* = 1), clear cell carcinoma (*n* = 1), osteoradionecrosis (*n* = 1), ameloblastoma (*n* = 2), and pleomorphic adenoma (*n* = 1).

**Table 1:** Baseline demographics and clinical characteristics.

	All patients (*n* = 42) n (%)	Maxillary reconstruction (*n* = 29) n (%)	Mandibular reconstruction (*n* = 13) n (%)	*P*-value
**Age**(years)	63 [22–78]	61 [22–78]	67 [35–78]	0.19
**Gender**				0.063
Male	25 (60)	20 (69)	5 (38)	
Female	17 (40)	9 (31)	8 (62)	
**WHO**				**0.025**
0	39 (93)	29 (100)	9 (75)	
1	3 (7)	0 (0)	3 (25)	
**CCI**				**0.011**
0	8 (19)	7 (24)	1 (8)	
1	5 (12)	4 (14)	1 (8)	
2	14 (33)	11 (38)	3 (23)	
3	7 (17)	5 (17)	2 (15)	
4	4 (10)	1 (3)	3 (23)	
5	4 (10)	1 (3)	3 (23)	
**ASA classification**				0.060
1	7 (17)	7 (24)	0	
2	24 (57)	16 (55)	8 (62)	
3	11 (26)	6 (21)	5 (38)	
**Smoker**				0.40
Never	21 (50)	16 (55)	5 (38)	
Previous	13 (31)	9 (31)	4 (31)	
Active	8 (19)	4 (14)	4 (31)	
**Previous ipsilateral neck surgery**				1.0
Yes	1 (2)	1 (3)	0	
No	41 (98)	28 (97)	13 (100)	
**Radiation therapy**				0.65
Previous RT	4 (10)	2 (7)	2 (15)	
Post-operative	29 (69)	21 (72)	8 (62)	
N/A	9 (21)	6 (21)	3 (23)	
**Chemotherapy**				
Previous	1 (2)	1 (3)	0	
Post-operative	5 (12)	3 (10)	2 (15)	
N/A	36 (86)	25 (86)	11 (85)	
**Surgical indication**				0.58^a^
**Malignant disease**				
Total	38 (90)	27 (93)	11 (85)	
T1	1 (2)	1 (3)	0 (0)	
T2	4 (10)	3 (10)	1 (8)	
T3	5 (12)	5 (17)	0 (0)	
T4	28 (67)	18 (62)	10 (77)	
**Benign disease**	4 (10)	2 (7)	2 (15)	

Values are expressed as number (%) or median [range].

N/A, not applicable; WHO, World Health Organisation performance status; CCI, Charlson comorbidity index; ASA, The American Society of Anaesthesiologists.

a Fischer's exact test calculated for malignant vs benign disease.

As seen in Table 1, there were no significant differences between the maxillary and mandibular groups regarding age, smoking history, treatment with (c)RT or surgical indication. All patients had good WHO performance status (WHO 0–1) and rather few comorbidities, with CCI scores of 0–5. However, the maxillary group had better WHO performance status (*p* = 0.025) and fewer comorbidities according to the CCI (*p* = 0.011) compared with the mandibular group.

All flaps, except for four, were harvested by the same senior plastic surgeon. All but one of the anastomoses were performed before flap inset.

Different types of SOFFs were harvested to suit the individual defects. All types of flaps, such as osseous flap, osseous flap with skin island or LD or trichemeric flaps, were used for maxillary and mandibular reconstructions. The osseous flap was the most common and used in 69 % (*n* = 29) of the cases (Table 2). The median duration of anaesthesia was approximately 13 h i.e., 802 min (range 591–1103 min) and the median surgical time was approximately 12 h i.e., 698 min (range 498–1022 min) for all patients with no significant difference between the maxillary and mandibular groups (Table 2).

**Table 2:** Intraoperative data.

	Maxillary reconstruction (*n* = 29) n (%)	Mandibular reconstruction (*n* = 13) n (%)	*P*-value
**Surgical access**			N/A
Weber Fergusson	13 (45)	0 (0)	
Submandibular	0 (0)	5 (38)	
Oral	16 (55)	0 (0)	
Submandibular/oral	0	8 (62)	
**SOFF**			0.41
Osseous flap only	22 (76)	7 (54)	
With skin island	3 (10)	4 (31)	
With LD	2 (7)	1 (8)	
With skin island and LD	2 (7)	1 (8)	
**Surgery**			
Duration of anaesthesia (min)	808 [623–1080]	800 [591–1103]	0.57
Duration of surgery (min)	706 [498–1008]	685 [529–1022]	0.97
Flap ischemia time (min)^a^	60 [46–226]	64 [54–115]	0.99
**Brown classification**^b^			N/A
I	1 (3)	N/A	
II	19 (66)	2 (8)	
III	8 (28)	N/A	
IV	1 (3)	N/A	
**Recipient artery**			**<0.001**
Superficial temporal artery	17 (59)	0	
Facial artery	12 (41)	12 (92)	
Superior thyroid artery	0	1 (8)	
**Recipient vein**^c^			**<0.001**
External jugular vein	1 (3)	5 (38)	
Facial vein	11 (38)	8 (62)	
Superficial temporal vein	17 (59)	0	

Values are expressed as number (%) or median [range]; N/A: not applicable; CCI: Charlson comorbidity index; LD: Latissimus dorsi muscle.

a Missing data for 8 patients i.e., maxillary group (*n* = 24) and mandibular group (*n* = 9).

b Two patients with mandibular reconstruction also had resections of the palate classified according to Brown classification.

c One patient in the maxillary group had an additional venous anastomosis. Two patients in the mandibular group had additional venous anastomoses.

Two patients in the mandibular group had a simultaneous resection involving the maxilla and were therefore given a Brown classification. Since the main resection and bony reconstruction were mandibular, they were classified in the mandibular reconstruction group. Type II, based on the Brown classification, was the most frequent defect in patients who underwent maxillary resection (19/31 patients).

There was a significant difference in the choice of vessel anastomoses, where the superficial temporal artery and vein were chosen as recipient vessels in 59 % (*n* = 17)) of the cases in the maxillary group while they were never used in the mandibular group (*p* < 0.001). The ischaemia time did not differ between the groups (Table 2).

### Postoperative outcome

As seen in Table 3, total flap failures were recorded in 21 % (*n* = 6) of cases in the maxillary group where two flaps were discarded perioperatively due to problems during the primary surgery. In one patient, the vessels’ anastomoses were planned to be performed after the bone flap inset, but the pedicle was lost after the inset. In another patient, the flap did not have adequate perfusion at the recipient site. Three of the six patients with total flap failures received a palatal obturator and three eventually underwent secondary reconstruction. No flaps were lost in the mandibular group (*p* = 0.076; Table 3). Overall, another 29 % (*n* = 9) of the 31 patients who underwent maxillary reconstruction developed oronasal fistula due to wound dehiscence or partial flap failure. Four patients with oronasal fistulas were eventually reconstructed with a radial forearm flap and one had a local facial artery myomucosal flap. One of the nine patients healed spontaneously. The three remaining patients were helped with palatal obturators.

**Table 3:** Post-operative outcomes.

	All patients (*n* = 42) n (%)	Maxillary reconstruction (*n* = 29) n (%)	Mandibular reconstruction (*n* = 13) n (%)	*P*-value
**Total flap failure**	6 (14)	6 (21)	0	0.076
**Palatinal oronasal fistula**^a^	9 (21)	8 (28)	1 (8)	N/A
**Complications according to Clavien–Dindo**				0.86
None	2 (5)	1 (3)	1 (8)	
Grade I	1 (2)	1 (3)	0	
Grade II	23 (55)	16 (55)	7 (54)	
Grade III	15 (36)	10 (35)	5 (39)	
Grade IV	1 (2)	1 (3)	0	
**Surgical take-backs**	10 (24)	7 (24)	3 (23)	1.0
**Post-operative hospitalization** (days)	16 [11–31]	15 [11–31]	19 [11–30]	0.099
Duration ICU (days)	1 (1–1)	1 (1–1)	1 (1–1)	0.64
**Donor site problems**				
Infection	2 (5)	1 (3)	1 (8)	0.53
Seroma	2 (5)	1 (3)	1 (8)	0.53
**Facial infection**	1 (2)	0	1 (8)	0.31
**Inadequate gingivobuccal sulcus**	6 (14)	3 (10)	3 (23)	0.35
**Aesthetic compromise**				
Overcontoured	7 (17)	4 (14)	3 (23)	0.66
Undercontoured	4 (10)	3 (10)	1 (8)	1.0
**Ectropion**	4 (10)	4 (14)	0	N/A
**Globe malposition**	3 (7)	3 (10)	0	N/A
**Surgical revision with new flap**				0.28
RFFF	4 (10)	4 (14)	0	
FAMM flap	1 (2)	1 (3)	0	
**Dental rehabilitation**				**0.038**
Dental implants	11 (26)	10 (34)	1 (8)	
Mobile prosthesis	4 (10)	4 (14)	0	
None	27 (64)	15 (52)	12 (92)	

Values are expressed as number (%) or median [range].

ICU, intensive care unit; RFFF, radial forearm free flap; FAMM flap, facial artery myomucosal flap.

a Two patients with bony mandibular reconstruction also had a resection of the palate that required a chimeric SOFF with soft tissue reconstruction of the palate. One of these patients developed a palatinal oronasal fistula.

There was no significant difference in general surgical complications between the maxillary and mandibular groups according to the Clavien–Dindo system. Grade II adverse effects were the most common and affected 55 % of the patients (*n* = 23), and were due to blood transfusion in all cases. In addition, 36 % (*n* = 15) of the patients received grade III Clavien–Dindo classification. Twelve of these cases were attributed to surgical takeback, of which three were a result of flap failure leading to additional surgery, one involved reintubation due to hypoxia, another was caused by an infected seroma at the donor site and one was due to a wound dehiscence of the lip that necessitated resuturing. Finally, one patient received a grade IV classification due to post-operative bowel obstruction leading to a bowel perforation requiring surgical intervention and additional intensive care.

There was no difference between the maxillary and mandibular groups in terms of similar rates of surgical takebacks, duration of ICU stay or length of hospitalisation (Table 3).

There were no significant differences between the groups concerning facial infections or inadequate gingivobuccal sulcus. In addition, there were no significant differences between the groups regarding aesthetic compromise, for both over- and under-contouring (Table 3).

In the maxillary group (*n* = 29), several eye-related problems were observed: 14 % (*n* = 4) had ectropion and globe malposition was observed in 10 % of the patients (*n* = 3), and one patient had both complications (Table 3). These adverse effects were associated with large tumour resections and all, but one patient, had a type III maxillary defect.

Patients in the maxillary group received dental rehabilitation with dental implants significantly more often than the patients in the mandibular group, 34 % (*n* = 10) vs. 8 % (*n* = 1; *p* *=* 0.038).

## Discussion

This study presents 42 consecutive SOFF cases performed at our institution, describing complications and post-operative sequelae. Several differences in procedure-specific complication rates were encountered depending on the reconstruction site. Total flap failure (14 %) and palatinal oronasal fistula formation (21 %) were only encountered in the maxillary group whereas patients in the mandibular group were spared from severe complications. In addition, eye-related problems (ectropion and globe malposition) were observed only in the maxillary group. On the other hand, more patients received dental rehabilitation in the maxillary group.

There are several identified risks factors eg., smoking, alcohol consumption, hypertension, coronary heart disease, diabetes, peripheral arterial vascular disease, renal failure and preoperative radiotherapy.^16^^,^^17^ Several of these risk factors are included in the CCI. We chose to specifically assess smoking and previous (c)RT; however, none of these factors were associated with flap failure in this study. Furthermore, the patients in the mandibular group had higher CCI scores. Nevertheless, all total flap failures were encountered in the maxillary group. The vessels chosen for anastomoses might be an explanation. Li et al. recommend the temporal vessels before cervical vessels for maxillary and midface reconstruction for aesthetic reasons and possibly for the lower complication rate.^18^ In our cohort, three total flap failures were observed with the facial artery as the recipient vessel and three total failures with the temporal artery as the recipient vessel.

Extended ischaemia time is not considered a cause for flap failure,^19^ although limited results are reported for SOFF failure and ischaemia time.^20^ In our series, the flap inset was performed after the vessel anastomoses and the ischaemia time was approximately one hour and consequently less likely to be the cause of the flap failures. However, flap inset after vessel anastomoses may also be the cause for pedicle compromise.

Maxillary reconstructions are challenging, and require 3D reconstruction with chimeric flaps. One of the more troublesome complications is the formation of oronasal fistulas.^7^ Oronasal formation after maxillary reconstruction varies from 4 to 21 % in different studies.^7^^,^^10^^,^^11^ Notably, in all these studies, several fistulas closed spontaneously (50–100 %) compared with 11 % of the oronasal fistulas in our series. In two of the studies, there were reports of whether the LD muscle was included, 18/25 (72 %) and 25/53 (47 %), respectively.^10^^,^^11^ In our cohort, the LD muscle was included in only 4/29 (14 %) of the maxillary cases, thus LD muscle use was sparse. However, the practice of enveloping the bone and reconstruction plate with an LD muscle has shown promising results in mandibular reconstructions^21^ and has subsequently been integrated into our standard institutional procedure, particularly for maxillary reconstructions.

For evident reasons, eye-related problems were only observed in the maxillary group. Our conclusion is that this issue is multifactorial and associated with a WF incision, the size of the tumour resection defect and subsequent radiotherapy. This is in line with the results presented by Sweeney et al.^22^

Han et al. suggested that the learning curve of free flap head and neck reconstruction appears to stabilise after approximately 20 cases.^23^ Accordingly, 67 % of our six SOFF total flap failures were among the first 20 maxillary reconstructions performed. Thus, the learning curve of this flap, might partly contribute to this high failure rate.

In our series, using the SOFF for mandibular reconstruction has been a safe and reliable option. This is in alignment with other studies where SOFF has been used successfully for mandibular reconstructions.^24^^,^^25^ The SOFF offers an excellent alternative to the FFF for bony reconstruction and for complex composite defects of the head and neck region with large soft tissue requirements, such as mandibular continuity defects with simultaneous intraoral and extraoral components.^26^, ^27^, ^28^ Moreover, in contrast to the FFF vessels, the SOFF vessels are typically unaffected by atherosclerosis.^29^^,^^30^

Gomez-Martinez de Lecea et al. recommend the use of the ipsilateral scapula tip flap for mandible angle reconstructions.^31^ The facial contour is affected by several factors, with the bony component being one part. In our opinion, dentoalveolar protrusion can adequately be addressed during dental rehabilitation and therefore the choice of flap harvest site should not be chosen solely on this indication.

Regarding general post-operative outcome, 23 (55 %) patients in our series received a grade II surgical complication grading according to the Clavien–Dindo-classification. Currently, our protocol states that the patients should have at least 100 g/L of haemoglobin post-operatively and consequently the reason that 23 patients had grade II complications was due to blood transfusion. Grades III and IV are classified as major complications, which occurred in 16/42 patients (38 %) in our study, which is in accordance with previous studies.^32^

In this study, 11 patients eventually received dental implants in their SOFF, with no documented failures despite having received RT; ten vs one patient in the maxillary and mandibular groups, respectively. The amount of bone was not a limiting factor in any of the other patients. It was either too early for dental rehabilitation, the patient did not require implantation, did not wish to undergo additional surgeries or had an ongoing malignancy. Possible reasons for only one patient in the mandibular group receiving dental implants might be poorer WHO performance status and higher comorbidity in the mandibular compared to the maxillary group. Moreover, the median age in the mandibular group was higher than in the maxillary group, though not significantly. Furthermore, the primary reason for choosing SOFF reconstruction in the mandibular group was not the possibility of dental rehabilitation, but rather that the FFF was not feasible and the option of maintaining the functionality of the jaw and facial contouring could be achieved with a SOFF.

There are continuing discussions on whether maxillectomy defects should be reconstructed or obturated, emphasising the need for personalised treatment plans and patient-related outcome measurements.^2^ A disadvantage of reconstruction is the prolonged operation time. Irawati et al. showed in their study that a prolonged operation time is significantly associated with an increased rate of surgical complications, an extended length of stay and a greater likelihood of return to the operating theatre.^33^

Not all patients with maxillary defects benefit from reconstruction with an osseous free flap. We consider a soft tissue free flap as the preferable choice in patients with significant comorbidities or posterior maxillary defects or in patients who are not eligible for dental rehabilitation. Correctly rebuilding the orbit, nasal and oral cavity is crucial for maintaining vision, the airway, oral competence and speech. The SOFF remains our first choice for extensive maxillary defects with a Brown classification of II-IV, where dental implants are warranted. However, studies on patient-related outcome measures are needed to better assess the patients’ perspective on maxillary reconstruction.

The constraints of our study are primarily its retrospective design, the limited number of patients, absence of a comparative control group and subjective aspects inherent in the assessment of aesthetic outcomes.

## Conclusion

The SOFF is an option for complex reconstruction of the maxilla but is associated with a relatively high rate of complications. In our study, some of the complications might be caused by an initial learning curve. Methods that can prevent or minimise sequelae such as oronasal fistulas for future patients are warranted. The SOFF is an excellent alternative for mandibular reconstructions.

## Conflicts of Interest

None to declare.
